# Body image perception and self-esteem in females with polycystic ovary syndrome: a systematic review and meta-analysis

**DOI:** 10.3389/fpsyg.2026.1755505

**Published:** 2026-03-27

**Authors:** Hyder Mirghani, Amani Shaman

**Affiliations:** 1 Department of Obstetrics and Gynecology, Faculty of Medicine, University of Tabuk, Tabuk, Saudi Arabia; 2 Department of Internal Medicine, Faculty of Medicine, University of Tabuk, Tabuk, Saudi Arabia

**Keywords:** body image, PCOS, perception, reproductive age, self-esteem

## Abstract

**Background:**

Polycystic ovary syndrome (PCOS) is a complex endocrine disorder associated with metabolic, reproductive, and psychosocial complications and affects 10% women of reproductive age worldwide. Evidence regarding its psychological impact, particularly body image perception and self-esteem, remains limited. This meta-analysis aimed to quantitatively evaluate differences in body image perception and self-esteem in women with PCOS and controls.

**Methods:**

A systematic search of PubMed, Web of Science, Cochrane Library, and Google Scholar was conducted from inception to December 30, 2025. Search terms included combinations of “PCOS” OR “polycystic ovary syndrome” AND “body image” OR “body perception” OR “self-esteem.” Observational studies assessing body image perception or self-esteem in women with PCOS compared with controls were eligible. Study selection and screening were performed according to predefined criteria. Pooled mean differences (MD) with 95% confidence intervals (CI) were calculated using meta-analytic models.

**Results:**

We identified 804 records, 31 full-text articles were screened, and 18 studies were included in the meta-analysis. Women with PCOS showed poorer body image perception compared with controls (MD = −0.94, 95% CI, −1.69 to −0.18, *p* = 0.02). In contrast, pooled analysis showed no statistically significant difference in self-esteem between women with PCOS and controls (MD = −0.50, 95% CI −1.29 to 0.29, *p* = 0.22).

**Conclusion:**

Women with PCOS experience poorer body image perception than healthy controls, with no difference in self-esteem. Integrating psychological assessment into PCOS care, and targeted interventions addressing body image concerns may be beneficial. Further large-scale studies across diverse populations using standardized assessment tools are warranted.

## Introduction

Polycystic ovary syndrome (PCOS) is a multifaceted syndrome with metabolic, fertility, and psychosocial concerns. The disease is common, and 8–13% of women of reproductive age are affected globally. The prevalence of PCOS is predicted by the interplay between genetic and environmental factors, and the diagnostic criteria (Almhmoud et al., 2024; Dunaif, 2011).

PCOS poses a significant burden through its physical and psychological symptoms (hirsutism, menstrual irregularities, infertility, anxiety, depression, and metabolic consequences) (Lentscher and Decherney, 2021). In addition, PCOS is associated with poor quality of life driven by weight gain, hirsutism, and infertility. Furthermore, a change in appearance significantly impact social norms and sexual relationships, leading to social withdrawal (AlAhmari et al., 2024; Kaushansky et al., 2017). The above physical and mental symptoms could precipitate reduced body appreciation, initiating a vicious cycle with deleterious consequences, and the current guidelines stressed the importance of mental health and its risk factors in women with PCOS (Jannink et al., 2025).

The 2023 International Evidence-Based Guideline updates for PCOS highlight *body image* as a newly recommended component of routine assessment in PCOS care (Teede et al., 2023). Body image encompasses an individual’s interpretation and evaluation of their appearance, size, physical functioning, perceived health, and sexual embodiment (Kertechian and El-Farr, 2025). It represents a multidimensional construct shaped by cognitive, emotional, and behavioral responses to one’s body (Alkheyr et al., 2024). The meta-analysis in novel because we included recent studies from 2024–2025 that were not available in previous meta-analyses.

Importantly, the development of body image and related self-esteem is strongly influenced by sociocultural norms. Consequently, the body-related attitudes of women living with PCOS are likely to differ between cultural contexts (Veale et al., 2016). There is a piece of evidence that cultural setting significantly shapes how individuals perceive and relate to their bodies (Swami et al., 2023; Stevens et al., 1994).

As negative body image intensifies, many women report heightened self-criticism and social withdrawal, which can evolve into broader self-esteem difficulties that significantly impact emotional well-being and quality of life (Khan et al., 2026). Additionally, PCOS common symptoms, including acne, hirsutism, and obesity, mediate the relationship between body image and self-esteem (Bazarganipour et al., 2013). Results from recent research pointed out that the lower self-esteem scores among women with PCOS are largely mediated by body image dissatisfaction. In addition, dermatological care, exercise, and mental health support significantly improve self-esteem levels over time (Davitadze et al., 2023; Magnusson et al., 2025). Because of this, holistic PCOS management increasingly includes psychological support to help individuals rebuild a positive sense of self (Alur-Gupta et al., 2024).

Despite the continuous evaluation and updates by the European Society of Human Reproduction and Embryology, women with PCOS often receive inadequate mental-health care relative to the recent recommendations. The consultation frequency and patient satisfaction are inadequate. These findings underscore a pressing need to strengthen the quality and accessibility of psychological support within PCOS services (Sourouni et al., 2024). Because of that, studies evaluating the body image in women with PCOS are highly relevant. We aimed to systematically review and quantitatively synthesize the available evidence on psychological outcomes related to body perception in women with PCOS. Specifically, the study evaluated differences in body image perception and self-esteem between women with PCOS and healthy controls.

## Subject and methods

This meta-analysis was conducted between November and December 2025 and followed the Preferred Reporting Items for Systematic Reviews and Meta-Analyses (PRISMA) guidelines. The review protocol was not registered in PROSPERO because most of the eligible studies were observational.

### Eligibility criteria according to PICOS

Studies were considered eligible if they met the following criteria:

Population: Women with PCOS according to recognized diagnostic criteria (Rotterdam, NIH, or similar).
Study Design: Clinical trials, prospective cohort studies, cross-sectional studies, or case–control studies from inception to December 30, 2025 were eligible. While, retrospective studies, case reports, case series, editorials, and opinions were excluded.
Outcomes: Studies assessing body image and/or self-esteem in women with PCOS and using validated quantitative assessment tools. Studies were included if they evaluated at least one of these outcomes because the aim of the review was to synthesize the available evidence regarding psychological aspects of body perception and self-worth in women with PCOS. When available, both outcomes were extracted and analyzed separately.
Comparator: Studies including a control group of women without PCOS were eligible.
Language and time-frame: Articles published in English from database inception to December 30, 2025.

### Information sources and search strategy

A comprehensive literature search was conducted independently by two reviewers (HM and AS) in the following electronic databases: PubMed/MEDLINE, Web of Science, Cochrane Library, and Google Scholar. The search was performed between November 1, 2025, and December 30, 2025. The search strategy included combinations of the following keywords and Medical Subject Headings (MeSH) terms: “polycystic ovary syndrome” OR “PCOS” AND “body image” OR “body perception” OR “body dissatisfaction” OR “body esteem” AND “self-esteem” OR “self esteem” OR “psychological well-being.” Boolean operators AND and OR were used to combine search terms. Reference lists of relevant articles were also manually screened to identify additional studies.

### Study selection

**Figure 1:**
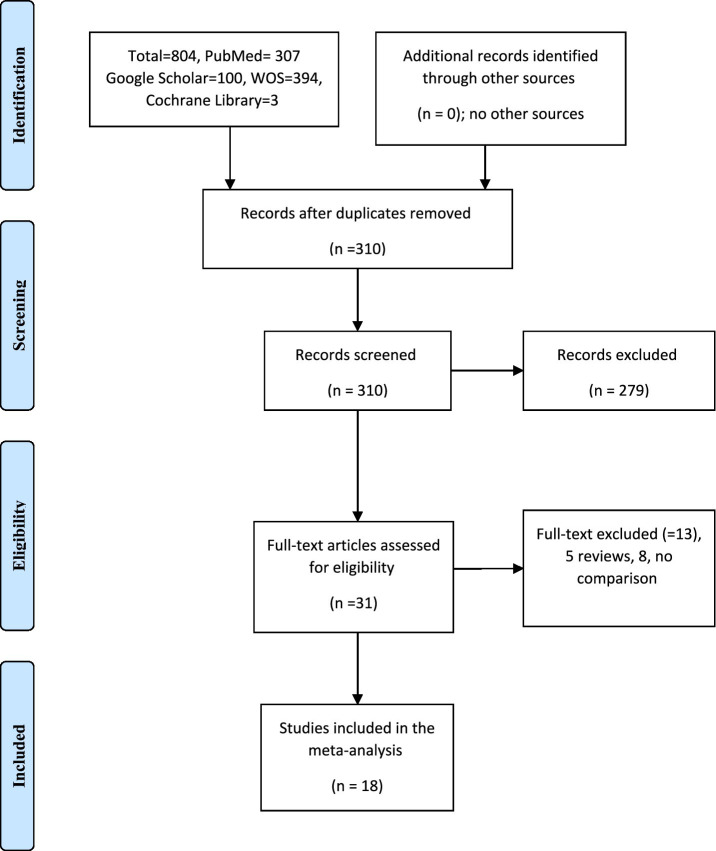
Body image and self-esteem in women with PCOS (The PRISMA chart).

All identified records were imported into a reference management software and duplicates were removed. Titles and abstracts were independently screened by two reviewers to identify potentially eligible studies. Full texts of relevant articles were subsequently assessed for eligibility. Disagreements between reviewers were resolved through discussion and consensus. A total of 708 records were identified through database searching. After removing duplicates, 307 unique records remained. Following title and abstract screening, 31 full-text articles were assessed for eligibility. Of these, 18 studies met the inclusion criteria and were included in the final meta-analysis. The study selection process is summarized in the PRISMA flow diagram (Figure 1).

### Data extraction

Data extraction was performed independently by two reviewers using a standardized data extraction form. The following information was collected from each study: first author, year of publication, country, study design, sample size, participants’ age, body mass index (BMI), diagnostic criteria for PCOS, outcome measures used for body image and/or self-esteem, and reported outcome data. Extracted data were compiled in Microsoft Excel (Tables 1, 2).

**Table 1:** Age, BMI, body image, and self-esteem in females with PCOS.

Author	Country	Study design	Sample size (PCOS /Control)	Age /years (PCOS)	Age/years (control)	BMI/kg/m^2^ (PCOS)	BMI kg/m^2^ (control)	Body image (PCOS)	Body image (control)	Self-esteem (PCOS)	Self-esteem (control)
Aba and Aytek Şik (2022)	Turkey	Case–control	97 vs. 95	28.23 ± 4.56	29.33 ± 5.61	25.08 ± 4.3	22.34 ± 3.74	132.11 ± 19.44	133.35 ± 21	Not reported	Not reported
Alur-Gupta et al. (2019)	USA	Cross-sectional	189 VS. 225	29.3 ± 14.65	32.1 ± 15.1	32.1 ± 19.65	33.9 ± 18.55	3.32 ± 0.76	3.34 ± 0.84	Not reported	Not reported
Amirshahi et al. (2024)	Iran	Trial	15 vs. 15	15–45	15–45	Not reported	Not reported	12.8.67 ± 8.86	140.06 ± 12.06	Not reported	Not reported
Annagür et al. (2014)	Turkey	Cross-sectional	83 vs. 64	22.27 ± 1.84	22.85 ± 2.06	23.85 ± 4.67	22.00 ± 2.43	97.62 ± 20.44	93.14 ± 17.13	Not reported	Not reported
Azizi Kutenaee et al. (2020)	Iran	Cross-sectional	201 vs. 199	27.86 ± 5.84	28.06 ± 6.51	22.73 ± 9.62	23.95 ± 4.96	39.17 ± 32.23	32.61 ± 11.11	15.32 ± 2.04	15.66 ± 1.90
Bafghi et al. (2024)	Iran	Case–control	30 vs. 30	27.63 ± 6.82	26.43 ± 5.43	25.1 ± 4.9	25.42 ± 4.15	222.23 ± 29.1	230.53 ± 29.1	Not reported	Not reported
Deeks et al. (2011)	Australia	Case–control	177 vs. 109	32.8 ± 7.8	41.9 ± 15.4	Not reported	Not reported	3.18 ± 0.74	3.18 ± 0.78	Not reported	Not reported
Doğan and Demir Çaltekin (2021)	Turkey	Case–control	51 vs. 50	24 ± 10	25 ± 8	22.7 ± 1.33	23 ± 1.12	73.12 ± 4.42	90.5 ± 11.12	Not reported	Not reported
Geller et al. (2025)	Israel	Cross-sectional	197 vs. 119	29.5 ± 5.3	33.3 ± 6.9	26.5 ± 6.1	23.3 ± 4.1	29.5 ± 8.6	24.4 ± 7.9	Not reported	Not reported
Himelein and Thatcher (2006)	USA	Case–control	40 vs. 60	Not reported	Not reported	Not reported	Not reported	3.11 ± 0.75	3.24 ± 0.78	Not reported	Not reported
Jannink et al. (2024)	Netherlands	Cross-sectional	502 vs. 523	30.8 ± 3.7	33.4 ± 3.8	26.7 ± 5.7	24.9 ± 4.3	33.0 ± 8.3	35.7 ± 7.6	Not reported	Not reported
Karacan et al. (2014)	Turkey	Cross-sectional	42 vs. 52	19.1 ± 2.3	19.7 ± 2.1 t	Not reported	Not reported	15.66 ± 5.53	16.1 ± 6.26	Not reported	Not reported
Moradi et al. (2020)	Iran	Trial	26 vs. 26	18–45	18–45	Not reported	Not reported	40.84 ± 13.50	44.07 ± 9.71	28.34 ± 5.69	29.11 ± 3.37
Pastore et al. (2011)	USA	Cross-sectional	94 vs. 96	18–44	18.44	28.77 ± 3.45	Not reported	32.03 ± 7.9	33.9 ± 9.37	Not reported	Not reported
Shereda et al. (2025)	Egypt	Trial	60	18–35	18–35	56.7% obese	53.3% obese	46.5 ± 12.4	45.6 ± 13.6	Not reported	Not reported
Zachurzok et al. (2021)	Poland	Cross-sectional	27 vs. 27	16.7 ± 1.2	16.1 ± 1.1	Not reported	Not reported	28.3 ± 4.6	25.0 ± 7.1	28.3 ± 4.6	25 ± 7.1
Çoban et al. (2019)	Turkey	Case–control	28 vs. 31	16.7 ± 1.1	16.4 ± 1.3	26.3 ± 4.2	21.1 ± 2.55	Not reported	Not reported	1.19 ± 0.93	1.52 ± 0.93
Sari et al. (2020)	Turkey	Case–control	50 vs. 37	16.01 ± 1.19	16.00 ± 1.49	26.18 ± 5.55	22.07 ± 4.05	Not reported	Not reported	18.44 ± 5.38	21.67 ± 4.24

BMI, body mass index; PCOS, polycystic ovary syndrome; vs., versus.

**Table 2:** Body image and self-esteem assessment tools.

Author	Body image diagnosis	Self-esteem diagnosis
Aba and Aytek Şik (2022)	Body Image Scale	Self-esteem
Alur-Gupta et al. (2019)	Multidimensional Body-Self Relations-Appearance Subscale (MBSRQ-AS), Stunkard Figure Rating Scale	Not assessed
Annagür et al. (2014)	Body Image Scale	Rosenberg Self-Esteem Scale
Azizi Kutenaee et al. (2020)	Body Image Concern Investigation	Rosenberg Self-esteem Scale
Bafghi et al. (2024)	Multidimensional Body-Self Relations Questionnaire	Not assessed
Deeks et al. (2011)	Multidimensional Body-Self Relations Questionnaire Appearance Scale; Body Features Satisfaction	Not assessed
Doğan and Demir Çaltekin (2021)	Body Awareness Questionnaire	Not assessed
Geller et al. (2025)	Body Appreciation (BAS-2), Body Dissatisfaction (EDI-BD)	Not assessed
Himelein and Thatcher (2006)	Multidimensional Body-Self Relations Questionnaire Appearance Scale; Body Features Satisfaction	Not assessed
Jannink et al. (2024)	Body Appreciation Scale-2 (BAS-2)	Not assessed
Karacan et al. (2014)	Stunkard Figure Rating Scale; Body Esteem Scale	Not assessed
Moradi et al. (2020)	Littleton’s development of the body image concern inventory	Rosenberg Self-Esteem Scale
Pastore et al. (2011)	Self-Report, Body Esteem Scale	Not assessed
Shereda et al. (2025)	Body Self-Image Questionnaire-Short Form (BSIQSF)	Not assessed
Zachurzok et al. (2021)	Not assessed	Body-Esteem Scale (BES)
Amirshahi et al. (2024)	Body Image Questionnaire	Not assessed
Çoban et al. (2019)	Not assessed	Rosenberg Self-Esteem Scale (RSES)
Sari et al. (2020)	Not assessed	Rosenberg Self-Esteem Scale

#### Body image and self-esteem assessment tools

Body image measures varied significantly between studies (Body Image Questionnaire, Body Self-Image Questionnaire-Short Form, Body Self-Image Questionnaire-Short Form, Littleton development of the body image concern inventory, Stunkard Figure Rating Scale; Body Esteem Scale, Body Appreciation Scale-2, Multidimensional Body-Self Relations Questionnaire Appearance Scale; Body Features Satisfaction, Body Image Concern Investigation, and Body Image Scale. Self-esteem was measured by the Rosenberg Self-Esteem Scale and the Body-Esteem Scale.

#### The quality of evidence

Analysis of the quality of evidence by Grading of Recommendations Assessment, Development, and Evaluation (GRADE) (Table 3).

**Table 3:** Analysis of the quality of evidence by Grading of Recommendations Assessment, Development, and Evaluation (GRADE).

Outcome	Number of the included studies	Study design of the included studies	Risk of bias of the included studies	Inconsistency (heterogeneity)	Indirectness	Imprecision	Other considerations	Overall clarity of evidence
Body image	16	Cross-sectional = 8, case–control = 5, trials = 3	Serious	Serious (*I*^2^ = 92%)	Not serious	Not serious	None	Low
Self-esteem	5	Cross-sectional = 2, case–control = 2, trials = 1	Serious	Serious (*I*^2^ = 69%)	Not serious	Not serious	None	Low

#### The quality of the included studies’ assessment

We used the Cochrane Risk of Bias (Sterne et al., 2019), and Newcastle–Ottawa Assessment Tools (Stang, 2010) (Tables 4, 5).

**Table 4:** The risk of bias of the included studies (Newcastle–Ottawa Scale).

Author	Selection	Comparability	Outcome/exposure	Total score
Aba and Aytek Şik (2022)	3	2	3	8
Alur-Gupta et al. (2019)	3	2	3	8
Annagür et al. (2014)	2	2	3	7
Azizi Kutenaee et al. (2020)	3	2	3	8
Bafghi et al. (2024)	2	2	3	7
Deeks et al. (2011)	2	2	3	7
Doğan and Demir Çaltekin (2021)	3	2	3	8
Geller et al. (2025)	2	2	3	7
Himelein and Thatcher (2006)	2	2	3	7
Jannink et al. (2024)	3	2	3	8
Karacan et al. (2014)	3	2	3	8
Pastore et al. (2011)	3	2	3	8
Zachurzok et al. (2021)	2	2	3	7
Çoban et al. (2019)	3	2	3	8
Sari et al. (2020)	2	2	3	7

**Table 5:** Risk of bias assessment of the included studies according to the Cochrane risk of bias of randomized controlled trials.

Author	Bias arising from the randomization process	Bias due to deviations from intended interventions	Bias due to missing outcome data	Bias in measurement of the outcome	Bias in selection of the reported result	Overall risk of bias
Moradi et al. (2020)	Low	High	High	Low	Some concerns	Some concern
Shereda et al. (2025)	Some concern	High	High	Low	Some concerns	Some concern
Amirshahi et al. (2024)	Low	High	High	Low	Some concerns	Some concern

The Newcastle–Ottawa Scale is a quality assessment tool used to evaluate the methodological quality of non-randomized studies. It assesses studies across three domains: selection of study groups, comparability of groups, and ascertainment of exposure or outcome. Studies are awarded a maximum of nine stars, with higher scores indicating better methodological quality and lower risk of bias. The Cochrane Risk of Bias Tool 2 (RoB 2) is a standardized tool developed by the Cochrane Collaboration to assess the risk of bias in randomized controlled trials. It evaluates bias across five domains: bias arising from the randomization process, deviations from intended interventions, missing outcome data, measurement of the outcome, and selection of the reported result. Each domain is rated as low risk of bias, some concerns, or high risk of bias, leading to an overall risk-of-bias judgment for each study.

### Statistical analysis

Review Manager version 5.4.1 (Cochrane Collaboration, Oxford, United Kingdom) was used for the data analyses. To analyze data focusing on body image and self-esteem in females with PCOS, forest plots were used to visualize and summarize the data. All the data were continuous, and the mean differences (MD) were used. A random effects meta-analysis was used to pool studies with similar characteristics, particularly when significant heterogeneity existed, and 95% confidence intervals (CIs) were calculated to quantify the precision of the pooled effect estimate. The *I*^2^ statistic was used to evaluate the degree of heterogeneity among studies. An *I*^2^ value <25% was considered low heterogeneity, whereas a value >50% indicated substantial heterogeneity. We generated funnel plots to assess potential publication bias in meta-analysis, including 10 or more studies. A subgroup analysis was conducted to identify the source of heterogeneity after removing studies contributing most to the heterogeneity. A *p-*value <0.05 was considered statistically significant.

## Results

### Characteristics of the included studies

We identified a total of 708 records. After removing duplicates, 307 unique articles remained. The titles and abstracts were screened for relevance, and 31 full-text articles were assessed for eligibility. Of these, 18 studies met the inclusion criteria and were included in the final meta-analysis. The study selection process is illustrated in the PRISMA flow diagram (Figure 1).

We included 18 studies which are published during 2006 to 2025. The studies were published in different countries, 11 from Asia, 3 from the USA, 3 from Europe, and one from Africa. The studies included 1909 patients with PCOS and 1818 controls. There were 7 case–control studies (Aba and Aytek Şik, 2022; Bafghi et al., 2024; Deeks et al., 2011; Doğan and Demir Çaltekin, 2021; Himelein and Thatcher, 2006; Çoban et al., 2019; Sari et al., 2020), 8 cross-sectional studies (Alur-Gupta et al., 2019; Annagür et al., 2014; Azizi Kutenaee et al., 2020; Geller et al., 2025; Jannink et al., 2024; Karacan et al., 2014; Pastore et al., 2011; Zachurzok et al., 2021), and 3 international studies (Amirshahi et al., 2024; Moradi et al., 2020; Shereda et al., 2025). Their ages ranged from 15 to 41.9 ± 15.4 years, and the BMI ranged from21.1 ± 2.55 to 33.9 ± 18.55.

### Body image perception

**Figure 2:**
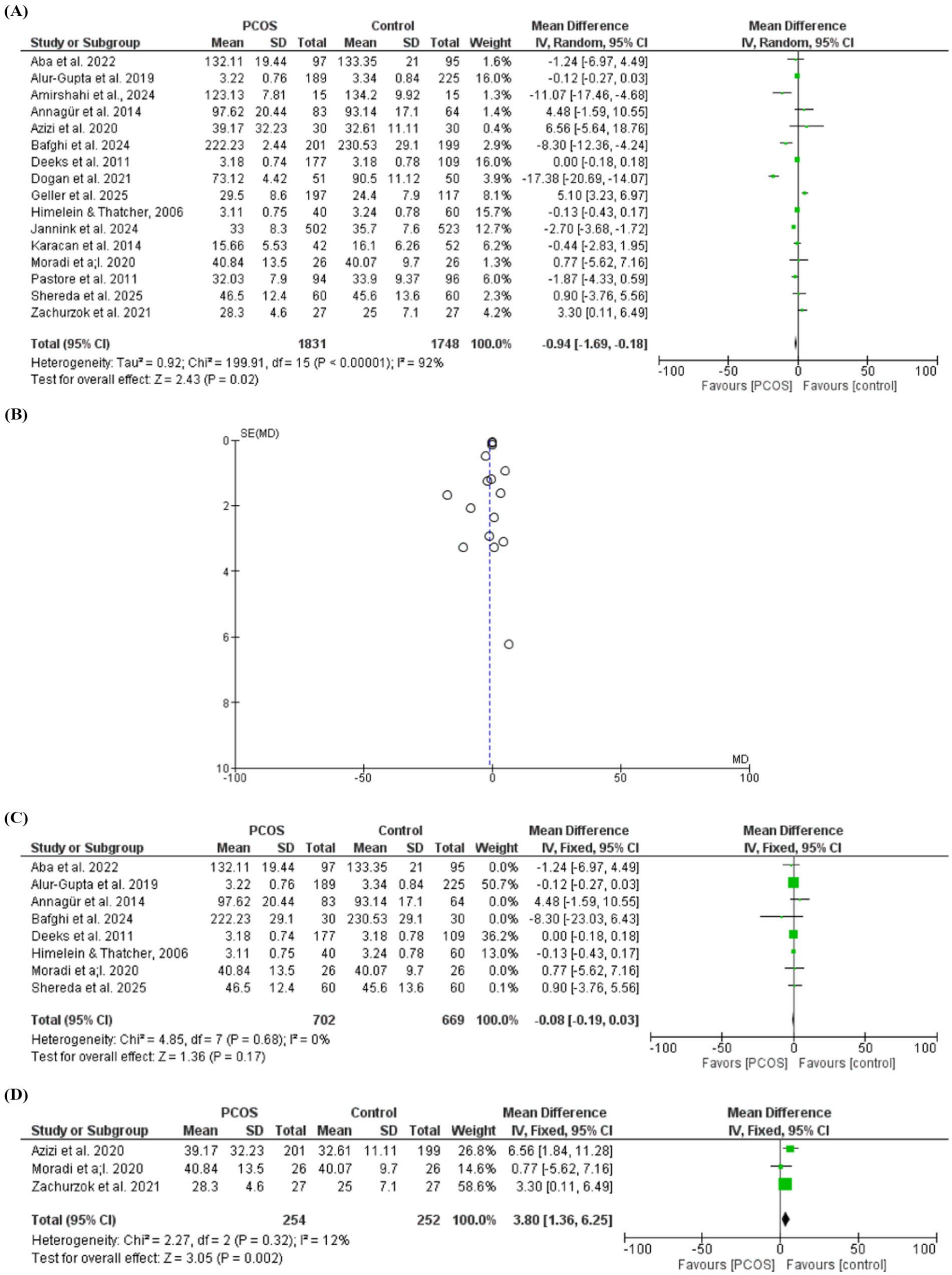
**(A)** Body image perception in women with PCOS and controls (forest plot). **(B)** Body image perception in women with PCOS and controls (funnel plot). **(C)** Body image perception in women with PCOS and controls after removing studies with a high contribution to heterogeneity. **(D)** Body image perception in women with PCOS and controls after removing studies with a high contribution to heterogeneity.

Sixteen studies evaluated body image perception in females with PCOS compared with controls. The pooled analysis showed that body image perception was significantly lower in females with PCOS [mean difference (MD) = −0.94, 95% CI: −1.69 to −0.18; *Z* = 2.43; *p* = 0.02]. However, substantial heterogeneity was observed among studies (chi-square = 199.91, *I*^2^ = 92%, *p* < 0.001). A subgroup analysis was conducted by removing studies that contributed substantially to heterogeneity. After exclusion of these studies, the difference between groups was no longer statistically significant (MD = −0.08, 95% CI: −0.19 to 0.03; *Z* = 1.36; *p* = 0.17), and heterogeneity was markedly reduced (chi-square = 4.85, *I*^2^ = 0%, *p* = 0.68). Body image perception was lower in PCOS compared to control subject when including studies that evaluated both body image and self-esteem, MD = 3.80, 95% CI: 1.36 to 6.25; *Z* = 3.05; *p* = 0.002), and heterogeneity (chi-square = 2.27, *I*^2^ = 12%, *p* = 0.32) (Figures 2A–D).

### Self-esteem

Four studies assessed self-esteem in females with PCOS compared with controls. The pooled analysis showed no significant difference in self-esteem between the two groups (MD = −0.50, 95% CI: −1.29 to 0.29; *Z* = 1.24; *p* = 0.22). Moderate heterogeneity was observed across studies (chi-square = 12.91, *I*^2^ = 69%, *p* = 0.01). In a subgroup analysis including studies that evaluated both body image perception and self-esteem, the results were not different between PCOS patients and control, (MD = 0.29, 95% CI: 1.54 to 2.13; *Z* = 0.31; *p* = 0.75). Moderate heterogeneity was observed across studies (chi-square = 1.62, *I*^2^ = 60%, *p* = 0.08).

**Figure 3:**
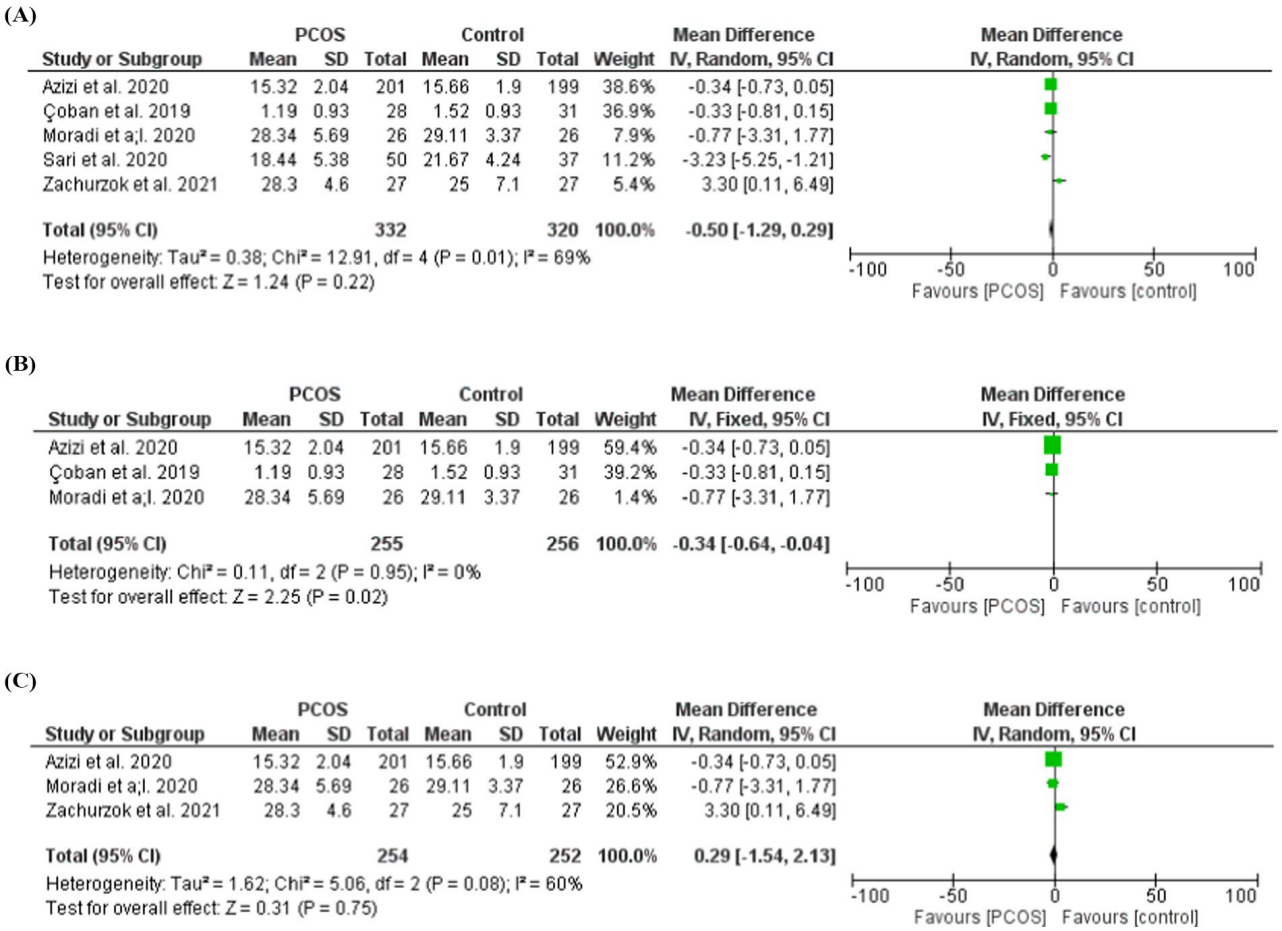
**(A)** Self-esteem in women with PCOS and controls. **(B)** Self-esteem in women with PCOS and controls after removing studies with a high contribution to heterogeneity. **(C)** Self-esteem in women with PCOS and controls (studies which evaluated both body image and self-esteem are included).

Following a subgroup analysis and removal of studies contributing to heterogeneity, self-esteem was found to be significantly higher in women with PCOS compared with controls (MD = −0.34, 95% CI: −0.64 to −0.04; *Z* = 2.35; *p* = 0.02), with no remaining heterogeneity (chi-square = 0.11, *I*^2^ = 0%, *p* = 0.95) (Figures 3A–C).

## Discussion

In this meta-analysis, we found a lower body image perception in females with PCOS compared to their counterparts without the syndrome. Self-esteem was not different between the two groups in the primary analysis, MD: −0.53, 95% CI, −1.27 to 0.21, and MD: −0.50, 95% CI, −1.29 to 0.29, respectively. However, self-esteem was higher in women with PCOS compared to controls after removing studies with a significant contribution to heterogeneity, MD: −0.34, 95% CI, −0.64 to −0.04, *p*-value = 0.02. The current findings were in line with Davitadze et al. (2023) who conducted a meta-analysis in which they included five studies and found low body perception in women with PCOS. Li et al. (2024) conducted a meta-analysis in women with PCOS and found no difference in self-esteem in females with PCOS and the control group, in agreement with the current findings.

Our findings supported the previous observations. However, Li et al. (2024) included only three studies in adolescent women with PCOS. While Davitadze et al. (2023) included only five studies investigating body image perception. In subgroup analyses, we found low self-esteem in women with PCOS after removing studies with high contribution too heterogeneity, MD: −0.34, 95% CI, −0.64 to −0.04, and no significant statistical differences were found regarding body image perception after removing studies with significant heterogeneity. Age and country could explain the different findings: young females might be more concerned about their body image, which predisposes them to depression and psychiatric disorders, including low self-esteem (Sari et al., 2020; Hofmann et al., 2025).

Females with PCOS frequently experience low self-esteem and negative body image due to the combined impact of clinical symptoms, hormonal influences, and sociocultural pressures. The clinical symptoms are largely driven by hyperandrogenism and insulin resistance, leading to hirsutism, acne, and alopecia (Jannink et al., 2025). The weight gain is usually viewed as personal failure and shame and is consistently associated with body dissatisfaction and psychological distress (Deeks et al., 2011; Hinchliff et al., 2012). These symptoms conflict with prevailing cultural beauty standards, intensifying appearance-related concerns and contributing to distorted body perception (Williams et al., 2024). In addition, endocrine disturbances and metabolic disturbances, including diabetes and metabolic syndrome in PCOS, are linked to elevated rates of anxiety and depression, which further undermine self-worth and amplify negative self-evaluations (Barry et al., 2011). Furthermore, reproductive challenges, including cycle irregularities and infertility fears, also play a significant role in shaping identity and feelings of inadequacy (Almhmoud et al., 2024). Importantly, women with PCOS are more prone to weight stigma, negative healthcare experiences, and misunderstanding of the condition. The above reasons reinforce internalized shame, creating a psychological environment that fosters poor self-esteem and persistent dissatisfaction with body image (Williams et al., 2015).

The heterogeneity observed in this meta-analysis could be explained by sociodemographic factors, the different methods used in body image perception, and self-esteem. In addition, PCOS is a multifaceted syndrome with different genotypes and diagnostic criteria (Azziz et al., 2006; Bizuneh et al., 2025).

The strength of this study is that we gave an up-to-date, broader insight into body image perception and self-esteem. We included recent studies from different regions worldwide.

### The study limitations

The results of this study should be viewed in the face of low levels of evidence. In addition, most of the studies were conducted in Asia. Therefore, generalization to the whole world cannot be ensured. Important limitations of this study are the majority of the included studies were observational, the heterogeneity in the measures used, and the fact that not all studies investigated body image and self-esteem together. In addition, we included studies reported either self-esteem, and/or body image perception.

### Clinical implications

It is essential to delineate the role of healthcare providers in operationalizing the 2023 guidelines recommending routine psychological screening for women with PCOS. Midwives, nurses, and gynecologists occupy a pivotal position at the frontline of care and are uniquely placed to integrate standardized psychological assessment into routine consultations. Systematic screening for body image disturbance, by using validated instruments, while also establishing clear referral pathways to mental health specialists. The role of nurses is vital by the administration of screening tools, monitoring symptom progression, providing psychoeducation, and reinforcing adherence to both medical and psychological interventions.

Midwives, can play an essential role in early identification of emotional vulnerability, and offering supportive counseling and continuity of care, interdisciplinary collaboration is highly required for an effective implementation, targeted training to enhance providers’ competence and confidence in discussing psychological distress, and the normalization of mental health conversations to reduce stigma.

Embedding self-esteem, and body image screening within routine PCOS care would foster a holistic, patient-centered model that acknowledges the bidirectional interplay between endocrine and psychological health, thereby improving both clinical outcomes and quality of life.

## Conclusion

Women with PCOS showed lower body image perception. While initial pooling showed no difference, sensitivity analysis suggests a significant reduction in self-esteem in specific PCOS populations. Careful patient selection for psychological intervention is suggested. Larger studies from different populations worldwide with objective assessment tools are needed.

## Data Availability

The original contributions presented in the study are included in the article/Supplementary material, further inquiries can be directed to the corresponding author.
