# Supplementary material for: Body image perception and self-esteem in females with polycystic ovary syndrome: a systematic review and meta-analysis
Source: Front Psychol. 2026 Mar 27;17:1755505. doi: 10.3389/fpsyg.2026.1755505 (PMC13066300; doi:10.3389/fpsyg.2026.1755505)
Supplement: Supplementary file 1 [file Table_1.DOC]

**Supplementary Table S1. Full search strategy used across databases**

| **Database** | **Search Strategy** |
| --- | --- |
| PubMed | (PCOS OR "Polycystic Ovary Syndrome" OR "Polycystic Ovarian Syndrome") AND ("Body Image" OR "Body Dissatisfaction" OR "Body Image Disturbance" OR "Body Perception") |
| Web of Science | TS=(PCOS OR "Polycystic Ovary Syndrome" OR "Polycystic Ovarian Syndrome") AND TS=("Body Image" OR "Body Dissatisfaction" OR "Body Image Disturbance" OR "Body Perception") |
| Google Scholar* | ("Polycystic Ovary Syndrome" OR PCOS) AND ("Body Image" OR "Body Dissatisfaction" OR "Body Image Disturbance") |
| Cochrane Library | (PCOS OR "Polycystic Ovary Syndrome") AND ("Body Image" OR "Body Dissatisfaction") |

For Google Scholar, the first 100 results sorted by relevance were screened due to database limitations.
